# Crystal structure of a 1:1 co-crystal of the anti­cancer drug gefitinib with azelaic acid

**DOI:** 10.1107/S2056989020006623

**Published:** 2020-05-22

**Authors:** Christy P. George, Ekta Sangtani, Rajesh G. Gonnade

**Affiliations:** a Physical and Materials Chemistry Division, CSIR-National Chemical Laboratory, Pune-411008, India; b Academy of Scientific and Innovative Research (AcSIR), Ghaziabad, Uttar Pradesh-201002, India

**Keywords:** anti­cancer, azelaic acid, co-crystal, crystal structure, gefitinib, hydrogen bond, π–π inter­actions

## Abstract

In the title co-crystal, C_22_H_24_ClFN_4_O_3_·C_9_H_16_O_4_, gefitinib (GTB; systematic name: quinazolin-4-amine) co-crystallizes with azelaic acid (AA; systematic name: nona­nedioic acid). The co-crystal has the monoclinic *P*2_1_/*n* centrosymmetric space group, containing one mol­ecule each of GTB and AA in the asymmetric unit. A structure overlay of the GTB mol­ecule in the co-crystal with that of its most stable polymorph revealed a significant difference in the conformation of the morpholine moiety. The significant deviation in the conformation of one of the acidic groups of azelaic acid from its usual linear chain structure could be due to the encapsulation of one acidic group in the pocket formed between the two pincers of GTB namely, the morpholine and phenyl moieties. Both GTB and AA mol­ecules form N—H⋯O, O—H⋯N, C—H⋯O hydrogen bonds with C—H⋯F close contacts along with off-stacked aromatic π–π inter­actions between the GTB mol­ecules.

## Abstract 2

Gefitinib, the anti­cancer drug, has been co-crystallized with azelaic acid to obtain a 1:1 co-crystal in the monoclinic *P*2_1_/*n* space group containing one mol­ecule each of gefitinib and azelaic acid in the asymmetric unit. Both mol­ecules are associated with each other through N—H⋯O, O—H⋯N, C—H⋯O and C—H⋯F hydrogen bonds.

## Chemical context   

Gefitinib (GTB, Iressa) is an orally administered chemotherapy treatment drug that inhibits tyrosine kinase (an enzyme that transports phosphates from ATP to the tyrosine residue of a protein) (Kobayashi & Hagiwara, 2013 ▸) for non-small-cell lung cancer (NSCLC), pancreatic cancer, breast cancer and several other types of cancer. Two polymorphs of GTB have been reported from our group previously, both of which crystallized in the triclinic *P* 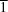 space group (Thorat *et al.*, 2014 ▸). The drug–drug co-crystal of GTB with furosemide has also been published (Thorat *et al.*, 2015 ▸). Some of the major side effects of GTB include rash, acne and dry skin. To overcome these after effects, there is a need for combination drug therapy. In this regard, we chose azelaic acid (AA), which is used for treating mild to moderate acne, both comedonal acne and inflammatory acne (Fitton & Goa, 1991 ▸). Furthermore, GTB is also known to form co-crystals with aliphatic di­carb­oxy­lic acids through N—H⋯O and O—H⋯N hydrogen bonds (Gonnade, 2015 ▸). AA is an aliphatic di­carb­oxy­lic acid (heptane-di­carb­oxy­lic acid), having seven CH_2_ groups in the alkyl chain. Two polymorphs of AA have been reported earlier, the *α* form is monoclinic, *P*2_1_/*c* (Caspari, 1928 ▸; Housty & Hospital, 1967 ▸) and the *β* form crystallizes in the monoclinic *C*2/*c* space group (Housty & Hospital, 1967 ▸). Both GTB and AA are non-volatile solids at room temperature and their respective melting points are in the ranges 192–195 K and 378–381 K.
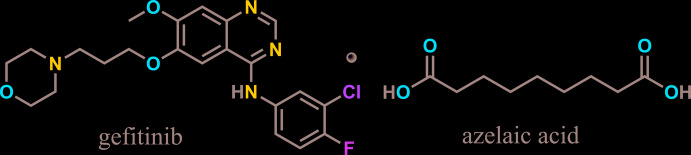


## Structural commentary   

**Figure 1:**
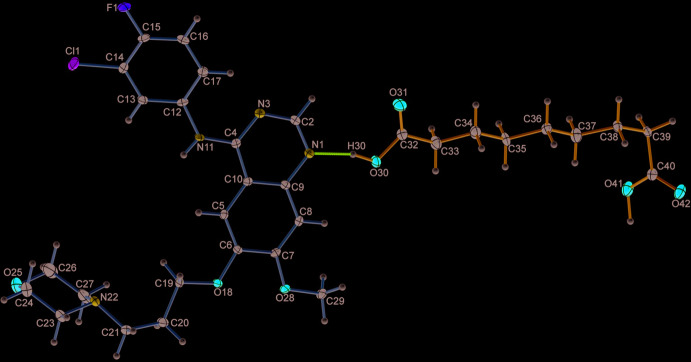
The asymmetric unit of the title compound, showing the atom labelling, 50% probability displacement ellipsoids for non-H atoms and hydrogen bonding with a dotted magenta line. H atoms are shown as small spheres of arbitrary radii.

**Figure 2:**
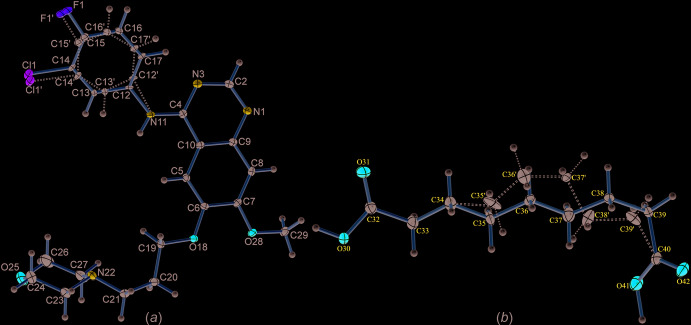
Crystal structures of GTB (*a*) and AA (*b*) in the co-crystal showing positional disorder of the halophenyl ring and alkyl chain, respectively.

**Figure 3:**
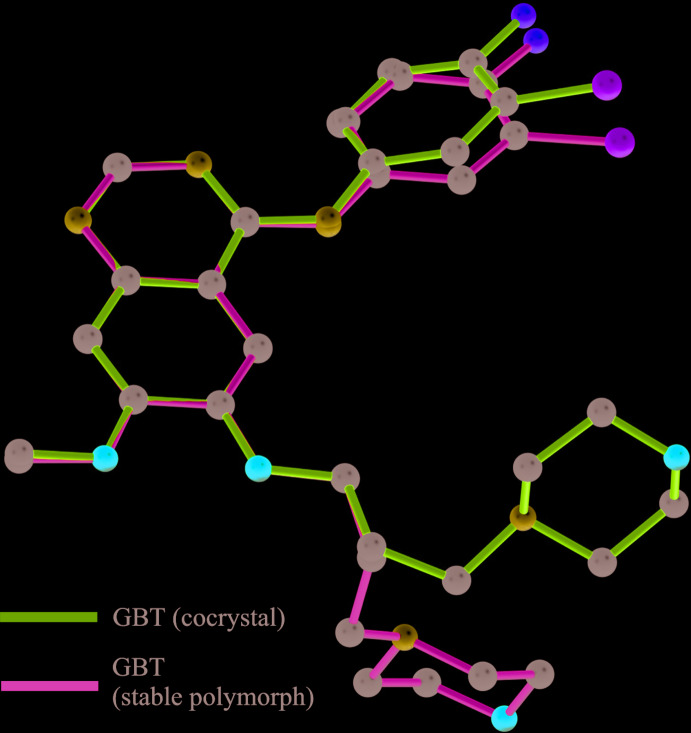
Structure overlay of GTB mol­ecule in the co-crystal (magenta) and its stable polymorph (green).

**Figure 4:**
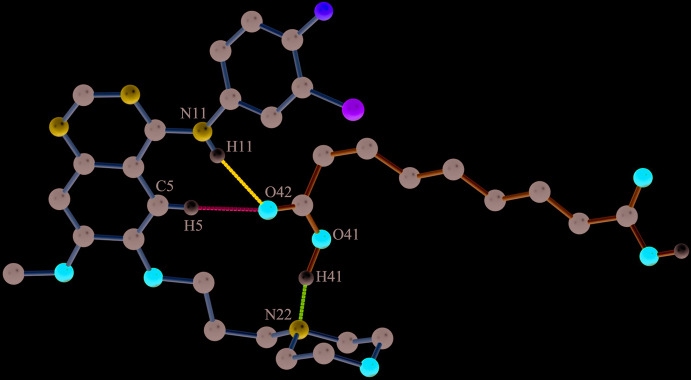
The ‘mol­ecular clip’-like geometry of GTB that accommodates a carboxyl group of AA. The mol­ecules inter­act through N—H⋯O, O—H⋯N and C—H⋯O hydrogen bonds.

The title compound GTB–AA (1:1) crystallizes in the monoclinic *P*2_1_/*n* centrosymmetric space group containing one mol­ecule of each in the asymmetric unit (Fig. 1 ▸, Table 1 ▸) (CCDC reference No. 2002536). The halophenyl ring of GTB and the alkyl (–CH_2_–) chain of AA exhibit positional disorder over two conformations, due to the free rotation around the N—C and C—C single bonds, respectively (Fig. 2 ▸ *a* and 2*b*). A structure overlay of the GTB mol­ecule based on a fit of the quinazoline groups in the co-crystal structure with that of its stable polymorph [the crystal structure of the stable polymorph of GTB was retrieved from the Cambridge Structural Database (Groom *et al.*, 2016 ▸), refcode: FARRUM02; Thorat *et al.*, 2014 ▸] revealed a considerable difference in the orientation of the morpholine moiety [torsion angles, C19—C20—C21—N22 = 54.0 (2)° for GTB in the co-crystal while the corresponding torsion angle in the stable polymorph of GTB is 74.3 (2)°] because of the conformationally flexible –CH_2_– spacer (Fig. 3 ▸). Whereas the conformation of the phenyl group showed a slight difference with a dihedral angle of 14.1 (2)° (the angular difference between the planes of halophenyl ring of both structures). The quinazoline, morpholine and phenyl moieties of GTB have acquired a roughly planar geometry in the co-crystal [torsion angle C12—C5—C19—N22 = 14.4 (2)°, only the N atom of morpholine is considered and not the full fragment], whereas in the stable polymorph of GTB, the morpholine moiety deviates significantly from the plane [the corresponding torsion angle is −75.7 (2)°]. The approximate planarity of the phenyl, quinazoline and morpholine (only N atom considered) moieties of GTB in the co-crystal seems to be due to the engagement of these groups with one of the acid groups of AA *via* N—H⋯O and O—H⋯N hydrogen bonds. The conformation of this acid group of AA shows a considerable departure from its usual linear chain structure due to an acquired bend at the 7th carbon atom (C39) [torsional difference 105.15 (19)° from the other end of the acid group, torsion angles, C32—C33—C34—C35 = −174.15 (19)° and C37—C38—C39—C40 = −69.0 (3)°]. The conformational bend could be due to the inclusion of the acid moiety in the pocket formed between the morpholine and phenyl moieties (which have a mol­ecular clip-like geometry) of GTB and the subsequent involvement of the carbonyl and hydroxyl groups of the included acid moiety in the formation of the N—H⋯O and O—H⋯N hydrogen bonds with the distantly located amine N—H and the N atom of the morpholine moiety, respectively (Fig. 4 ▸). The other acid group of AA forms an O—H⋯N hydrogen bond with the N atom of the quinazoline moiety.

## Supra­molecular features   

**Figure 5:**
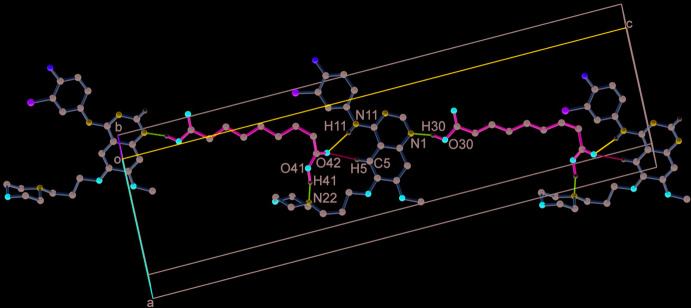
A one-dimensional chain formed by GTB and AA mol­ecules along the *ac* diagonal *via* O—H⋯N, N—H⋯O and C—H⋯O hydrogen bonds.

**Figure 6:**
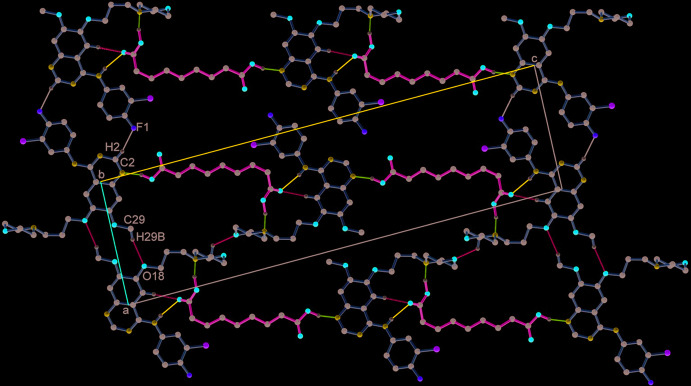
Two-dimensional layered assembly of GTB and AA along the *ac* diagonal. The neighbouring one-dimensional chains are stitched through C—H⋯F and C—H⋯O hydrogen bonds.

**Figure 7:**
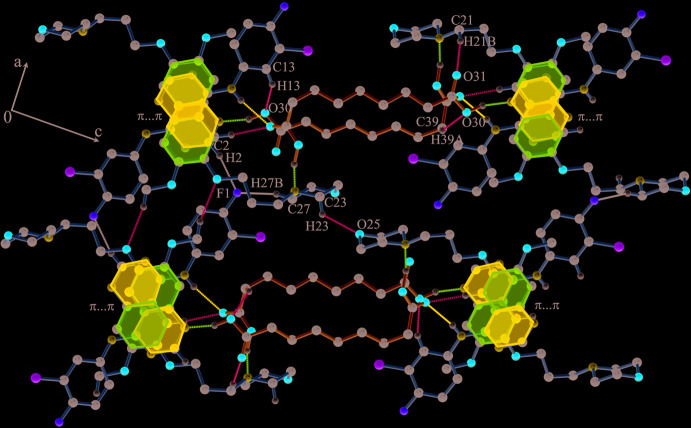
The view of the mol­ecular packing along the *b* axis showing the association of GTB mol­ecules through aromatic π–π inter­actions along with C—H⋯F and C—H⋯O inter­actions.

The closely associated mol­ecules of GTB and AA (through an O30—H30⋯N1 hydrogen bond) constitute a ‘zero-dimensional’ supra­molecular motif wherein a carboxyl OH of AA donates its H atom to the quinazoline N atom (Fig. 1 ▸). Adjacent *n*-glide symmetry-related ‘zero-dimensional’ motifs are linked firmly along the *ac* diagonal by strong N—H⋯O, O—H⋯N and C—H⋯O hydrogen bonds to generate a one-dimensional linear chain structure (Fig. 5 ▸, Table 1 ▸). The cavity created by GTB as a result of its ‘mol­ecular clip’-like geometry encapsulates the other carb­oxy­lic acid group of AA. In the cavity, the carboxyl oxygen (O42) accepts the H atoms from amine N11—H11 and C5—H5 to form N11—H11⋯O42^i^ and C5—H5⋯O42^i^ hydrogen bonds (symmetry operations are given in the footnote to Table 1 ▸). In turn, the carboxyl OH (O41—H41) of AA donates its H atom to the morpholine N22 to make a O41—H41⋯N22^ii^ hydrogen bond. The neighbouring anti­parallel chains are stitched centrosymmetrically through C2—H2⋯F1^iii^ contacts and C29—H29*B*⋯O18^iv^ hydrogen bonds to form a two-dimensional layered assembly in the *ac* plane (Fig. 6 ▸). A view of the mol­ecular packing down the *b* axis reveals the stacking of the 2D layers by aromatic π–π inter­actions between centrosymmetrically related quinazoline rings [inter­planar spacing, 3.396 (13) Å] (*Cg*2⋯*Cg*2^vii^, *Cg*2⋯*Cg*3^vii^, *Cg*2⋯*Cg*3^viii^ and *Cg*3⋯*Cg*3^vii^; *Cg*2 is the centroid of the N1/C2/N3/C4/C10/C9 ring and *Cg*3 is the centroid of the C5–C10 ring, Table 1 ▸). Mol­ecules between the two layers are also connected by C27—H27*B*⋯F1^vi^ contacts and C23—H23*B*⋯O25^v^, C21—H21*B*⋯O31^vii^, C13—H13⋯O30^viii^ and C39—H39*A*⋯O30^ix^ hydrogen bonds to generate the three-dimensional packing (Fig. 7 ▸, Table 1 ▸).

## Database survey   

A search for the title co-crystal in the Cambridge Structural Database (CSD, Version 5.41, the update of March 2020; Groom *et al.*, 2016 ▸) found no hits. However, searches for GTB and AA gave 8 and 35 hits, respectively. A search for the GTB mol­ecule showed that the amine N—H moiety is involved in N—H⋯O hydrogen-bond formation either with the morpholine oxygen in both of its polymorphs (Thorat *et al.*, 2014 ▸) or with the water oxygen (Gilday *et al.*, 2005 ▸; Thorat *et al.*, 2015 ▸). For the AA search, 17 hits were found only for its two polymorphs (refcodes: AZELAC01–AZELAC17) wherein the AA mol­ecules are found to be associated by the conventional dimeric O—H⋯O hydrogen bonds (Caspari, 1928 ▸; Housty & Hospital, 1967 ▸). The remaining hits were for either co-crystals with amides (Tothadi & Phadkule, 2019 ▸; Thompson *et al.*, 2011 ▸; Karki *et al.*, 2009 ▸), pyridines (Braga *et al.*, 2010 ▸; Martins *et al.*, 2016 ▸; Krueger *et al.*, 2017 ▸) or complexes with Ni (Zhao *et al.*, 2012 ▸), Fe (Braga *et al.*, 2006 ▸) or Ba (Grzesiak *et al.*, 2012 ▸).

## Synthesis and crystallization   

Co-crystallization was carried out using equimolar amounts of commercial samples of GTB and AA by grinding combined with a slow evaporation method. The grinding experiment was performed manually using a mortar and pestle. The 1:1 stoichiometric molar ratio of GTB (45 mg, 0.1 mmol) and AA (19 mg, 0.1 mmol) was ground for about 15 minutes using dry (neat) grinding. The ground sample was dissolved in *n*-butanol and heated for ∼10 minutes to ensure the complete dissolution of the sample. The solution was filtered into the crystallization flask to remove the impurity and undissolved compound, and the solution was allowed to evaporate at room temperature (298–300 K). Elongated needle-shaped colourless crystals were obtained after 1–2 h. The melting point of the obtained co-crystal was 398–399 K.

## Refinement   

Crystal data, data collection and structure refinement details are summarized in Table 2 ▸. All H atoms (except for hy­droxy and amine H atoms) were placed in geometrically idealized positions, with C—H = 0.95 Å for phenyl H atoms, C—H = 0.99 Å for methyl­ene H atoms and C—H = 0.98 Å for methyl H atoms. They were constrained to ride on their parent atoms, with *U*_iso_(H) = 1.2*U*_eq_(C) for phenyl and methyl­ene, and 1.5*U*_eq_(C) for methyl groups. The O- (O30) and N-bound H atoms were located in difference-Fourier maps and refined isotropically. However, the O-bound H atom was placed in a geometrically idealized positions using HFIX 148 as the O—H distance was longer when refined with its located position in the difference-Fourier map. It was constrained to ride on its parent atom (O41), with *U*_iso_(H) = 1.5*U*_eq_(O). The long O—H distance could be due to its involvement in the strong O—H⋯N hydrogen-bond formation with N22. The difference *F*_o_–*F*_c_ map shows that the H atom could be residing part of the time on O41 and part of the time on N22.

## Supplementary Material

Crystal structure: contains datablock(s) I. DOI: 10.1107/S2056989020006623/pk2627sup1.cif

Structure factors: contains datablock(s) I. DOI: 10.1107/S2056989020006623/pk2627Isup2.hkl

Supporting information file. DOI: 10.1107/S2056989020006623/pk2627Isup3.cml

CCDC reference: 2002536

Additional supporting information:  crystallographic information; 3D view; checkCIF report

## Figures and Tables

**Table 1:** Hydrogen-bond geometry (Å, °)

*D*—H⋯*A*	*D*—H	H⋯*A*	*D*⋯*A*	*D*—H⋯*A*
N11—H11⋯O42^i^	0.84 (2)	2.20 (2)	3.025 (2)	169.6 (18)
O41—H41⋯N22^ii^	1.01	1.78	2.6566 (19)	144
O30—H30⋯N1	0.99 (3)	1.65 (3)	2.6135 (19)	166 (2)
C5—H5⋯O42^i^	0.95	2.25	3.194 (2)	170
C2—H2⋯F1^iii^	0.95	2.15	3.07 (3)	163
C2—H2⋯F1′^iii^	0.95	2.32	3.253 (3)	166
C29—H29*B*⋯O18^iv^	0.98	2.65	3.6101 (19)	167
C23—H23*B*⋯O25^v^	0.99	2.57	3.220 (2)	123
C27—H27*B*⋯F1^vi^	0.99	2.71	3.68 (4)	166
C39—H39*A*⋯O30^vii^	0.99	2.50	3.255 (4)	133
C21—H21*B*⋯O31^viii^	0.99	2.29	3.234 (2)	160
C13—H13⋯O30^ix^	0.95	2.39	3.139 (3)	135
C13′—H13′⋯O30^ix^	0.95	2.53	3.268 (3)	135
*Cg*2⋯*Cg*2^viii^			3.5358 (11)	0 (1)
*Cg*2⋯*Cg*3^viii^			3.7909 (11)	1 (1)
*Cg*2⋯*Cg*3^ix^			3.7530 (11)	1 (1)
*Cg*3⋯*Cg*3^viii^			3.7934 (11)	0 (1)

Symmetry codes: (i) 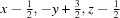; (ii) 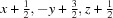; (iii) 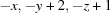; (iv) 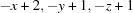; (v) 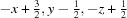; (vi) 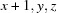; (vii) 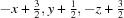; (viii) 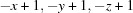; (ix) 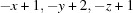.

**Table 2:** Experimental details

Crystal data	
Chemical formula	C_22_H_24_ClFN_4_O_3_·C_9_H_16_O_4_
*M*_r_	635.12
Crystal system, space group	Monoclinic, *P*2_1_/*n*
Temperature (K)	100
*a*, *b*, *c* (Å)	10.7716 (10), 7.4153 (13), 38.175 (7)
β (°)	92.311 (5)
*V* (Å^3^)	3046.7 (8)
*Z*	4
Radiation type	Mo *K*α
μ (mm^−1^)	0.19
Crystal size (mm)	0.28 × 0.19 × 0.04

Data collection	
Diffractometer	Bruker D8 VENTURE Kappa Duo PHOTON II CPAD
Absorption correction	Multi-scan (*SADABS*; Bruker, 2016 ▸)
*T*_min_, *T*_max_	0.950, 0.993
No. of measured, independent and observed [*I* > 2σ(*I*)] reflections	92337, 7338, 5413
*R*_int_	0.162
(sin θ/λ)_max_ (Å^−1^)	0.661

Refinement	
*R*[*F* ^2^ > 2σ(*F* ^2^)], *wR*(*F* ^2^), *S*	0.045, 0.110, 1.02
No. of reflections	7338
No. of parameters	473
No. of restraints	126
H-atom treatment	H atoms treated by a mixture of independent and constrained refinement
Δρ_max_, Δρ_min_ (e Å^−3^)	0.37, −0.32

Computer programs: *APEX3* (Bruker, 2016 ▸), *SAINT-Plus* (Bruker, 2016 ▸), *SHELXS97* (Sheldrick, 2008 ▸), *SHELXL2014/7* (Sheldrick, 2015 ▸), *ORTEP-3* (Farrugia, 2012 ▸), **Mercury* 2020.1* (Macrae *et al.*, 2020 ▸), *SHELXTL* (Sheldrick, 2008 ▸), *PLATON* (Spek, 2020 ▸), *publCIF* (Westrip, 2010 ▸).

## supplementary crystallographic information

### Crystal data

**Table:**

C_22_H_24_ClFN_4_O_3_·C_9_H_16_O_4_	*F*(000) = 1344
*M**_r_* = 635.12	*D*_x_ = 1.385 Mg m^−^^3^
Monoclinic, *P*2_1_/*n*	Mo *K*α radiation, λ = 0.71073 Å
*a* = 10.7716 (10) Å	Cell parameters from 9964 reflections
*b* = 7.4153 (13) Å	θ = 2.5–36.4°
*c* = 38.175 (7) Å	µ = 0.19 mm^−^^1^
β = 92.311 (5)°	*T* = 100 K
*V* = 3046.7 (8) Å^3^	Thin Needle, colourless
*Z* = 4	0.28 × 0.19 × 0.04 mm

### Data collection

**Table:**

Bruker D8 VENTURE Kappa Duo PHOTON II CPAD diffractometer	7338 independent reflections
Radiation source: micro-focus sealed tube, Incoatech IµS HB	5413 reflections with *I* > 2σ(*I*)
Multilayer mirrors monochromator	*R*_int_ = 0.162
Detector resolution: 7.39 pixels mm^-1^	θ_max_ = 28.0°, θ_min_ = 2.4°
φ and ω scans	*h* = −14→14
Absorption correction: multi-scan (SADABS; Bruker, 2016)	*k* = −9→9
*T*_min_ = 0.950, *T*_max_ = 0.993	*l* = −50→50
92337 measured reflections	

### Refinement

**Table:**

Refinement on *F*^2^	126 restraints
Least-squares matrix: full	Hydrogen site location: mixed
*R*[*F*^2^ > 2σ(*F*^2^)] = 0.045	H atoms treated by a mixture of independent and constrained refinement
*wR*(*F*^2^) = 0.110	*w* = 1/[σ^2^(*F*_o_^2^) + (0.0458*P*)^2^ + 1.2854*P*] where *P* = (*F*_o_^2^ + 2*F*_c_^2^)/3
*S* = 1.02	(Δ/σ)_max_ = 0.001
7338 reflections	Δρ_max_ = 0.37 e Å^−^^3^
473 parameters	Δρ_min_ = −0.32 e Å^−^^3^

### Special details

**Table:**

Geometry. All esds (except the esd in the dihedral angle between two l.s. planes) are estimated using the full covariance matrix. The cell esds are taken into account individually in the estimation of esds in distances, angles and torsion angles; correlations between esds in cell parameters are only used when they are defined by crystal symmetry. An approximate (isotropic) treatment of cell esds is used for estimating esds involving l.s. planes.

### Fractional atomic coordinates and isotropic or equivalent isotropic displacement parameters (Å^2^)

**Table:**

	*x*	*y*	*z*	*U*_iso_*/*U*_eq_	Occ. (<1)
N1	0.47579 (12)	0.8204 (2)	0.55135 (4)	0.0133 (3)	
C2	0.36066 (14)	0.8699 (2)	0.54311 (5)	0.0148 (4)	
H2	0.3136	0.9129	0.5619	0.018*	
N3	0.30169 (12)	0.8670 (2)	0.51143 (4)	0.0144 (3)	
C4	0.36644 (14)	0.8072 (2)	0.48478 (4)	0.0113 (3)	
C5	0.56600 (14)	0.6768 (2)	0.46286 (4)	0.0111 (3)	
H5	0.5318	0.6671	0.4396	0.013*	
C6	0.68614 (14)	0.6245 (2)	0.47037 (4)	0.0109 (3)	
C7	0.73862 (13)	0.6397 (2)	0.50508 (4)	0.0116 (3)	
C8	0.66847 (14)	0.7041 (2)	0.53159 (4)	0.0117 (3)	
H8	0.7033	0.7129	0.5548	0.014*	
C9	0.54411 (14)	0.7573 (2)	0.52418 (4)	0.0115 (3)	
C10	0.49287 (14)	0.7456 (2)	0.48986 (4)	0.0108 (3)	
N11	0.31164 (12)	0.8064 (2)	0.45205 (4)	0.0129 (3)	
H11	0.3547 (17)	0.784 (3)	0.4348 (5)	0.018 (5)*	
C12	0.1907 (2)	0.8603 (3)	0.44138 (7)	0.0084 (5)	0.915 (7)
C13	0.1724 (2)	0.9202 (3)	0.40659 (7)	0.0103 (5)	0.915 (7)
H13	0.2409	0.9296	0.3918	0.012*	0.915 (7)
C14	0.0538 (2)	0.9655 (3)	0.39399 (6)	0.0126 (5)	0.915 (7)
C15	−0.0455 (2)	0.9516 (5)	0.41588 (7)	0.0121 (6)	0.915 (7)
C16	−0.0294 (2)	0.8968 (3)	0.45021 (8)	0.0114 (5)	0.915 (7)
H16	−0.0984	0.8906	0.4649	0.014*	0.915 (7)
C17	0.0893 (2)	0.8503 (3)	0.46331 (6)	0.0103 (5)	0.915 (7)
H17	0.1014	0.8120	0.4870	0.012*	0.915 (7)
Cl1	0.03309 (6)	1.0446 (3)	0.35140 (2)	0.0239 (3)	0.915 (7)
F1	−0.1656 (19)	0.981 (6)	0.4079 (9)	0.0207 (5)	0.085 (7)
C12'	0.165 (3)	0.869 (4)	0.4489 (8)	0.0084 (5)	0.085 (7)
C13'	0.180 (3)	0.904 (3)	0.4171 (9)	0.0103 (5)	0.085 (7)
H13'	0.2607	0.8997	0.4082	0.012*	0.085 (7)
C14'	0.088 (3)	0.944 (3)	0.3964 (9)	0.0126 (5)	0.085 (7)
C15'	−0.034 (2)	0.956 (6)	0.4083 (11)	0.0121 (6)	0.085 (7)
C16'	−0.038 (3)	0.918 (4)	0.4387 (10)	0.0114 (5)	0.085 (7)
H16'	−0.1167	0.9307	0.4489	0.014*	0.085 (7)
C17'	0.051 (3)	0.863 (4)	0.4588 (8)	0.0103 (5)	0.085 (7)
H17'	0.0340	0.8158	0.4813	0.012*	0.085 (7)
Cl1'	0.0350 (8)	0.977 (3)	0.3483 (3)	0.0239 (3)	0.085 (7)
F1'	−0.15929 (17)	0.9946 (5)	0.40135 (6)	0.0207 (5)	0.915 (7)
O18	0.76536 (10)	0.55753 (17)	0.44664 (3)	0.0146 (3)	
C19	0.71844 (14)	0.5374 (3)	0.41114 (4)	0.0140 (3)	
H19A	0.6840	0.6529	0.4021	0.017*	
H19B	0.6523	0.4447	0.4096	0.017*	
C20	0.82895 (15)	0.4798 (3)	0.39031 (5)	0.0167 (4)	
H20A	0.8851	0.5844	0.3879	0.020*	
H20B	0.8755	0.3852	0.4036	0.020*	
C21	0.79365 (15)	0.4080 (2)	0.35412 (5)	0.0162 (4)	
H21A	0.8708	0.3825	0.3418	0.019*	
H21B	0.7492	0.2923	0.3567	0.019*	
N22	0.71453 (13)	0.5303 (2)	0.33193 (4)	0.0170 (3)	
C23	0.69993 (18)	0.4460 (3)	0.29657 (5)	0.0252 (4)	
H23A	0.6568	0.3287	0.2985	0.030*	
H23B	0.7829	0.4231	0.2873	0.030*	
C24	0.6265 (2)	0.5668 (4)	0.27160 (6)	0.0392 (6)	
H24A	0.6195	0.5091	0.2482	0.047*	
H24B	0.5415	0.5827	0.2801	0.047*	
O25	0.68388 (16)	0.7383 (3)	0.26857 (4)	0.0467 (5)	
C26	0.6932 (2)	0.8207 (3)	0.30238 (6)	0.0426 (6)	
H26A	0.6088	0.8361	0.3113	0.051*	
H26B	0.7306	0.9419	0.3002	0.051*	
C27	0.77011 (19)	0.7117 (3)	0.32823 (5)	0.0261 (4)	
H27A	0.8558	0.7002	0.3201	0.031*	
H27B	0.7740	0.7734	0.3512	0.031*	
O28	0.85921 (10)	0.58683 (17)	0.50862 (3)	0.0148 (3)	
C29	0.92077 (14)	0.6098 (3)	0.54230 (5)	0.0162 (4)	
H29A	0.9179	0.7371	0.5491	0.024*	
H29B	1.0075	0.5712	0.5412	0.024*	
H29C	0.8790	0.5367	0.5597	0.024*	
O30	0.58731 (10)	0.86845 (18)	0.61271 (3)	0.0181 (3)	
H30	0.533 (2)	0.852 (4)	0.5915 (7)	0.056 (8)*	
O31	0.41210 (11)	0.90780 (19)	0.64135 (4)	0.0238 (3)	
C32	0.52448 (15)	0.9063 (2)	0.64073 (5)	0.0169 (4)	
C33	0.60896 (16)	0.9548 (3)	0.67194 (5)	0.0209 (4)	
H33A	0.6142	1.0879	0.6735	0.025*	
H33B	0.6934	0.9093	0.6676	0.025*	
C34	0.57017 (16)	0.8827 (3)	0.70716 (5)	0.0200 (4)	
H34A	0.5747	0.7494	0.7073	0.024*	
H34B	0.4834	0.9184	0.7112	0.024*	
C35	0.6584 (3)	0.9608 (5)	0.73679 (8)	0.0196 (6)	0.770 (4)
H35A	0.7449	0.9243	0.7324	0.023*	0.770 (4)
H35B	0.6546	1.0941	0.7360	0.023*	0.770 (4)
C36	0.6250 (3)	0.8968 (3)	0.77317 (6)	0.0178 (6)	0.770 (4)
H36A	0.6289	0.7634	0.7740	0.021*	0.770 (4)
H36B	0.5388	0.9335	0.7777	0.021*	0.770 (4)
C37	0.7126 (2)	0.9744 (4)	0.80178 (8)	0.0186 (6)	0.770 (4)
H37A	0.7989	0.9407	0.7967	0.022*	0.770 (4)
H37B	0.7070	1.1077	0.8011	0.022*	0.770 (4)
C38	0.6847 (2)	0.9101 (3)	0.83854 (7)	0.0174 (6)	0.770 (4)
H38A	0.6933	0.7772	0.8394	0.021*	0.770 (4)
H38B	0.5973	0.9397	0.8432	0.021*	0.770 (4)
C39	0.7695 (3)	0.9931 (5)	0.86770 (8)	0.0150 (7)	0.770 (4)
H39A	0.7688	1.1260	0.8653	0.018*	0.770 (4)
H39B	0.7366	0.9625	0.8908	0.018*	0.770 (4)
C35'	0.6128 (9)	0.9559 (19)	0.7400 (3)	0.0196 (6)	0.230 (4)
H35C	0.6048	1.0888	0.7387	0.023*	0.230 (4)
H35D	0.7025	0.9281	0.7433	0.023*	0.230 (4)
C36'	0.5482 (8)	0.8921 (13)	0.7725 (2)	0.023 (2)	0.230 (4)
H36C	0.5562	0.7594	0.7743	0.027*	0.230 (4)
H36D	0.4586	0.9209	0.7698	0.027*	0.230 (4)
C37'	0.6007 (7)	0.9780 (13)	0.8072 (2)	0.023 (2)	0.230 (4)
H37C	0.5988	1.1109	0.8048	0.028*	0.230 (4)
H37D	0.5456	0.9453	0.8264	0.028*	0.230 (4)
C38'	0.7281 (8)	0.9218 (12)	0.8171 (3)	0.0139 (19)	0.230 (4)
H38C	0.7850	0.9748	0.8002	0.017*	0.230 (4)
H38D	0.7333	0.7890	0.8149	0.017*	0.230 (4)
C39'	0.7737 (12)	0.9740 (19)	0.8538 (3)	0.019 (2)	0.230 (4)
H39C	0.7657	1.1065	0.8559	0.023*	0.230 (4)
H39D	0.7161	0.9199	0.8705	0.023*	0.230 (4)
C40	0.90344 (15)	0.9248 (2)	0.86617 (5)	0.0153 (4)	
O41	0.97487 (11)	1.00506 (19)	0.84437 (3)	0.0235 (3)	
H41	1.060 (2)	0.950 (2)	0.8463 (4)	0.035*	
O42	0.93796 (11)	0.80413 (19)	0.88602 (3)	0.0224 (3)	

### Atomic displacement parameters (Å^2^)

**Table:**

	*U*^11^	*U*^22^	*U*^33^	*U*^12^	*U*^13^	*U*^23^
N1	0.0133 (6)	0.0141 (7)	0.0125 (7)	0.0010 (6)	0.0024 (5)	−0.0003 (6)
C2	0.0139 (7)	0.0173 (9)	0.0133 (8)	0.0029 (7)	0.0036 (6)	−0.0007 (7)
N3	0.0127 (6)	0.0165 (8)	0.0140 (7)	0.0020 (6)	0.0015 (5)	0.0003 (6)
C4	0.0119 (7)	0.0092 (8)	0.0128 (8)	−0.0007 (6)	0.0005 (6)	0.0004 (7)
C5	0.0106 (7)	0.0123 (8)	0.0101 (8)	−0.0005 (6)	−0.0005 (6)	0.0001 (7)
C6	0.0109 (7)	0.0106 (8)	0.0114 (8)	−0.0002 (6)	0.0027 (6)	0.0000 (7)
C7	0.0088 (7)	0.0100 (8)	0.0160 (9)	−0.0006 (6)	−0.0011 (6)	0.0015 (7)
C8	0.0126 (7)	0.0116 (8)	0.0107 (8)	−0.0007 (6)	−0.0023 (6)	0.0007 (7)
C9	0.0127 (7)	0.0090 (8)	0.0129 (8)	−0.0001 (6)	0.0020 (6)	−0.0001 (7)
C10	0.0112 (7)	0.0078 (8)	0.0135 (8)	0.0005 (6)	0.0012 (6)	0.0011 (6)
N11	0.0076 (6)	0.0195 (8)	0.0118 (7)	0.0034 (6)	0.0017 (5)	−0.0015 (6)
C12	0.0041 (10)	0.0098 (8)	0.0114 (13)	0.0021 (7)	0.0028 (7)	−0.0018 (8)
C13	0.0085 (8)	0.0165 (10)	0.0059 (12)	0.0021 (7)	0.0002 (9)	0.0017 (8)
C14	0.0071 (11)	0.0177 (10)	0.0127 (9)	0.0050 (8)	−0.0034 (9)	0.0015 (8)
C15	0.0086 (8)	0.0157 (9)	0.0120 (17)	0.0024 (7)	−0.0011 (7)	−0.0018 (11)
C16	0.0083 (8)	0.0165 (10)	0.0093 (12)	0.0008 (7)	0.0016 (9)	−0.0005 (9)
C17	0.0035 (10)	0.0140 (9)	0.0132 (9)	0.0011 (8)	−0.0014 (8)	0.0002 (7)
Cl1	0.01550 (19)	0.0375 (8)	0.0185 (3)	0.0082 (3)	−0.00091 (18)	0.0091 (4)
F1	0.0077 (5)	0.0352 (9)	0.0190 (13)	0.0063 (5)	−0.0013 (6)	−0.0007 (9)
C12'	0.0041 (10)	0.0098 (8)	0.0114 (13)	0.0021 (7)	0.0028 (7)	−0.0018 (8)
C13'	0.0085 (8)	0.0165 (10)	0.0059 (12)	0.0021 (7)	0.0002 (9)	0.0017 (8)
C14'	0.0071 (11)	0.0177 (10)	0.0127 (9)	0.0050 (8)	−0.0034 (9)	0.0015 (8)
C15'	0.0086 (8)	0.0157 (9)	0.0120 (17)	0.0024 (7)	−0.0011 (7)	−0.0018 (11)
C16'	0.0083 (8)	0.0165 (10)	0.0093 (12)	0.0008 (7)	0.0016 (9)	−0.0005 (9)
C17'	0.0035 (10)	0.0140 (9)	0.0132 (9)	0.0011 (8)	−0.0014 (8)	0.0002 (7)
Cl1'	0.01550 (19)	0.0375 (8)	0.0185 (3)	0.0082 (3)	−0.00091 (18)	0.0091 (4)
F1'	0.0077 (5)	0.0352 (9)	0.0190 (13)	0.0063 (5)	−0.0013 (6)	−0.0007 (9)
O18	0.0105 (5)	0.0228 (7)	0.0106 (6)	0.0042 (5)	−0.0001 (4)	−0.0030 (5)
C19	0.0114 (7)	0.0206 (9)	0.0098 (8)	0.0009 (7)	−0.0015 (6)	−0.0007 (7)
C20	0.0121 (7)	0.0224 (10)	0.0156 (9)	0.0028 (7)	0.0009 (6)	−0.0040 (8)
C21	0.0169 (8)	0.0157 (9)	0.0160 (9)	0.0040 (7)	0.0016 (7)	−0.0010 (7)
N22	0.0211 (7)	0.0188 (8)	0.0113 (7)	0.0053 (6)	0.0028 (6)	−0.0004 (6)
C23	0.0275 (9)	0.0347 (12)	0.0133 (9)	0.0098 (9)	0.0015 (7)	−0.0053 (9)
C24	0.0400 (12)	0.0615 (17)	0.0161 (10)	0.0193 (12)	0.0034 (9)	0.0049 (11)
O25	0.0590 (10)	0.0591 (12)	0.0235 (9)	0.0253 (9)	0.0179 (7)	0.0200 (8)
C26	0.0637 (15)	0.0314 (13)	0.0347 (14)	0.0212 (12)	0.0252 (12)	0.0158 (11)
C27	0.0396 (11)	0.0170 (10)	0.0228 (11)	0.0020 (8)	0.0143 (9)	0.0010 (8)
O28	0.0096 (5)	0.0212 (7)	0.0135 (6)	0.0038 (5)	−0.0025 (4)	−0.0022 (5)
C29	0.0123 (7)	0.0214 (10)	0.0145 (9)	0.0028 (7)	−0.0052 (6)	−0.0036 (7)
O30	0.0150 (5)	0.0276 (7)	0.0116 (6)	0.0001 (5)	−0.0004 (5)	−0.0023 (6)
O31	0.0141 (6)	0.0344 (8)	0.0232 (7)	−0.0014 (5)	0.0020 (5)	−0.0030 (6)
C32	0.0174 (8)	0.0181 (9)	0.0153 (9)	−0.0011 (7)	0.0011 (7)	0.0000 (7)
C33	0.0192 (8)	0.0276 (11)	0.0159 (9)	−0.0043 (8)	−0.0005 (7)	−0.0023 (8)
C34	0.0254 (9)	0.0197 (10)	0.0148 (9)	0.0011 (8)	−0.0001 (7)	−0.0006 (8)
C35	0.0201 (15)	0.0227 (11)	0.0159 (12)	−0.0061 (15)	0.0000 (13)	−0.0001 (9)
C36	0.0162 (13)	0.0209 (13)	0.0161 (12)	−0.0027 (10)	−0.0023 (10)	−0.0003 (10)
C37	0.0196 (12)	0.0229 (15)	0.0133 (14)	−0.0058 (11)	−0.0012 (11)	0.0028 (12)
C38	0.0125 (10)	0.0247 (14)	0.0149 (14)	−0.0037 (9)	−0.0005 (10)	0.0013 (10)
C39	0.0134 (11)	0.0207 (15)	0.0111 (16)	−0.0003 (10)	0.0009 (14)	−0.0017 (15)
C35'	0.0201 (15)	0.0227 (11)	0.0159 (12)	−0.0061 (15)	0.0000 (13)	−0.0001 (9)
C36'	0.014 (5)	0.035 (5)	0.019 (4)	0.002 (4)	0.001 (3)	−0.003 (4)
C37'	0.015 (4)	0.033 (5)	0.021 (5)	0.000 (3)	−0.002 (3)	−0.006 (4)
C38'	0.017 (4)	0.013 (4)	0.011 (5)	−0.002 (3)	−0.004 (4)	0.001 (4)
C39'	0.023 (4)	0.021 (5)	0.014 (6)	0.003 (3)	0.010 (5)	−0.005 (5)
C40	0.0153 (7)	0.0154 (9)	0.0148 (9)	−0.0042 (7)	−0.0028 (7)	−0.0027 (7)
O41	0.0185 (6)	0.0302 (8)	0.0215 (7)	−0.0046 (6)	−0.0012 (5)	0.0094 (6)
O42	0.0171 (6)	0.0288 (8)	0.0209 (7)	−0.0047 (5)	−0.0044 (5)	0.0085 (6)

### Geometric parameters (Å, º)

**Table:**

N1—C2	1.319 (2)	C24—O25	1.421 (3)
N1—C9	1.378 (2)	C24—H24A	0.9900
C2—N3	1.343 (2)	C24—H24B	0.9900
C2—H2	0.9500	O25—C26	1.428 (3)
N3—C4	1.333 (2)	C26—C27	1.499 (3)
C4—N11	1.360 (2)	C26—H26A	0.9900
C4—C10	1.442 (2)	C26—H26B	0.9900
C5—C6	1.370 (2)	C27—H27A	0.9900
C5—C10	1.417 (2)	C27—H27B	0.9900
C5—H5	0.9500	O28—C29	1.433 (2)
C6—O18	1.3631 (19)	C29—H29A	0.9800
C6—C7	1.424 (2)	C29—H29B	0.9800
C7—O28	1.3585 (18)	C29—H29C	0.9800
C7—C8	1.373 (2)	O30—C32	1.319 (2)
C8—C9	1.414 (2)	O30—H30	0.99 (3)
C8—H8	0.9500	O31—C32	1.212 (2)
C9—C10	1.404 (2)	C32—C33	1.513 (2)
N11—C12	1.408 (2)	C33—C34	1.521 (3)
N11—C12'	1.64 (3)	C33—H33A	0.9900
N11—H11	0.84 (2)	C33—H33B	0.9900
C12—C17	1.404 (3)	C34—C35'	1.426 (12)
C12—C13	1.407 (3)	C34—C35	1.559 (4)
C13—C14	1.389 (3)	C34—H34A	0.9900
C13—H13	0.9500	C34—H34B	0.9900
C14—C15	1.387 (3)	C35—C36	1.525 (4)
C14—Cl1	1.734 (3)	C35—H35A	0.9900
C15—F1	1.335 (19)	C35—H35B	0.9900
C15—C16	1.377 (3)	C36—C37	1.527 (4)
C16—C17	1.397 (3)	C36—H36A	0.9900
C16—H16	0.9500	C36—H36B	0.9900
C17—H17	0.9500	C37—C38	1.524 (4)
C12'—C13'	1.26 (4)	C37—H37A	0.9900
C12'—C17'	1.31 (3)	C37—H37B	0.9900
C13'—C14'	1.27 (4)	C38—C39	1.540 (4)
C13'—H13'	0.9500	C38—H38A	0.9900
C14'—C15'	1.41 (4)	C38—H38B	0.9900
C14'—Cl1'	1.92 (3)	C39—C40	1.533 (4)
C15'—C16'	1.20 (4)	C39—H39A	0.9900
C15'—F1'	1.395 (19)	C39—H39B	0.9900
C16'—C17'	1.27 (4)	C35'—C36'	1.522 (14)
C16'—H16'	0.9500	C35'—H35C	0.9900
C17'—H17'	0.9500	C35'—H35D	0.9900
O18—C19	1.435 (2)	C36'—C37'	1.556 (12)
C19—C20	1.519 (2)	C36'—H36C	0.9900
C19—H19A	0.9900	C36'—H36D	0.9900
C19—H19B	0.9900	C37'—C38'	1.469 (11)
C20—C21	1.515 (2)	C37'—H37C	0.9900
C20—H20A	0.9900	C37'—H37D	0.9900
C20—H20B	0.9900	C38'—C39'	1.516 (15)
C21—N22	1.486 (2)	C38'—H38C	0.9900
C21—H21A	0.9900	C38'—H38D	0.9900
C21—H21B	0.9900	C39'—C40	1.501 (13)
N22—C27	1.482 (2)	C39'—H39C	0.9900
N22—C23	1.490 (2)	C39'—H39D	0.9900
C23—C24	1.509 (3)	C40—O42	1.221 (2)
C23—H23A	0.9900	C40—O41	1.300 (2)
C23—H23B	0.9900	O41—H41	1.01 (2)
			
C2—N1—C9	116.13 (14)	O25—C26—C27	112.41 (18)
N1—C2—N3	128.05 (15)	O25—C26—H26A	109.1
N1—C2—H2	116.0	C27—C26—H26A	109.1
N3—C2—H2	116.0	O25—C26—H26B	109.1
C4—N3—C2	116.72 (14)	C27—C26—H26B	109.1
N3—C4—N11	118.80 (14)	H26A—C26—H26B	107.9
N3—C4—C10	121.53 (15)	N22—C27—C26	109.72 (18)
N11—C4—C10	119.66 (14)	N22—C27—H27A	109.7
C6—C5—C10	119.83 (15)	C26—C27—H27A	109.7
C6—C5—H5	120.1	N22—C27—H27B	109.7
C10—C5—H5	120.1	C26—C27—H27B	109.7
O18—C6—C5	125.13 (15)	H27A—C27—H27B	108.2
O18—C6—C7	114.31 (13)	C7—O28—C29	117.44 (13)
C5—C6—C7	120.56 (14)	O28—C29—H29A	109.5
O28—C7—C8	125.43 (15)	O28—C29—H29B	109.5
O28—C7—C6	114.31 (14)	H29A—C29—H29B	109.5
C8—C7—C6	120.26 (14)	O28—C29—H29C	109.5
C7—C8—C9	119.64 (15)	H29A—C29—H29C	109.5
C7—C8—H8	120.2	H29B—C29—H29C	109.5
C9—C8—H8	120.2	C32—O30—H30	112.8 (15)
N1—C9—C10	121.46 (14)	O31—C32—O30	124.29 (17)
N1—C9—C8	118.33 (15)	O31—C32—C33	123.51 (16)
C10—C9—C8	120.21 (14)	O30—C32—C33	112.16 (14)
C9—C10—C5	119.48 (14)	C32—C33—C34	115.76 (15)
C9—C10—C4	116.09 (14)	C32—C33—H33A	108.3
C5—C10—C4	124.43 (15)	C34—C33—H33A	108.3
C4—N11—C12	128.84 (17)	C32—C33—H33B	108.3
C4—N11—C12'	116.5 (11)	C34—C33—H33B	108.3
C4—N11—H11	119.4 (13)	H33A—C33—H33B	107.4
C12—N11—H11	111.4 (13)	C35'—C34—C33	123.6 (5)
C12'—N11—H11	123.9 (17)	C33—C34—C35	109.25 (18)
C17—C12—C13	119.82 (19)	C33—C34—H34A	109.8
C17—C12—N11	123.1 (3)	C35—C34—H34A	109.8
C13—C12—N11	117.1 (2)	C33—C34—H34B	109.8
C14—C13—C12	119.7 (2)	C35—C34—H34B	109.8
C14—C13—H13	120.2	H34A—C34—H34B	108.3
C12—C13—H13	120.2	C36—C35—C34	112.7 (2)
C15—C14—C13	119.6 (2)	C36—C35—H35A	109.1
C15—C14—Cl1	121.1 (2)	C34—C35—H35A	109.1
C13—C14—Cl1	119.2 (2)	C36—C35—H35B	109.1
F1—C15—C16	110.4 (15)	C34—C35—H35B	109.1
F1—C15—C14	127.9 (16)	H35A—C35—H35B	107.8
C16—C15—C14	121.6 (2)	C35—C36—C37	111.9 (2)
C15—C16—C17	119.50 (19)	C35—C36—H36A	109.2
C15—C16—H16	120.3	C37—C36—H36A	109.2
C17—C16—H16	120.3	C35—C36—H36B	109.2
C16—C17—C12	119.7 (2)	C37—C36—H36B	109.2
C16—C17—H17	120.1	H36A—C36—H36B	107.9
C12—C17—H17	120.1	C38—C37—C36	113.7 (2)
C13'—C12'—C17'	116 (3)	C38—C37—H37A	108.8
C13'—C12'—N11	89 (2)	C36—C37—H37A	108.8
C17'—C12'—N11	152 (3)	C38—C37—H37B	108.8
C12'—C13'—C14'	122 (3)	C36—C37—H37B	108.8
C12'—C13'—H13'	119.2	H37A—C37—H37B	107.7
C14'—C13'—H13'	119.2	C37—C38—C39	114.0 (2)
C13'—C14'—C15'	122 (3)	C37—C38—H38A	108.7
C13'—C14'—Cl1'	145 (3)	C39—C38—H38A	108.7
C15'—C14'—Cl1'	93 (2)	C37—C38—H38B	108.7
C16'—C15'—F1'	99 (3)	C39—C38—H38B	108.7
C16'—C15'—C14'	112 (3)	H38A—C38—H38B	107.6
F1'—C15'—C14'	149 (4)	C40—C39—C38	111.9 (2)
C15'—C16'—C17'	127 (3)	C40—C39—H39A	109.2
C15'—C16'—H16'	116.4	C38—C39—H39A	109.2
C17'—C16'—H16'	116.4	C40—C39—H39B	109.2
C16'—C17'—C12'	121 (3)	C38—C39—H39B	109.2
C16'—C17'—H17'	119.7	H39A—C39—H39B	107.9
C12'—C17'—H17'	119.7	C34—C35'—C36'	117.2 (8)
C6—O18—C19	117.35 (12)	C34—C35'—H35C	108.0
O18—C19—C20	105.66 (12)	C36'—C35'—H35C	108.0
O18—C19—H19A	110.6	C34—C35'—H35D	108.0
C20—C19—H19A	110.6	C36'—C35'—H35D	108.0
O18—C19—H19B	110.6	H35C—C35'—H35D	107.2
C20—C19—H19B	110.6	C35'—C36'—C37'	113.9 (8)
H19A—C19—H19B	108.7	C35'—C36'—H36C	108.8
C21—C20—C19	113.76 (13)	C37'—C36'—H36C	108.8
C21—C20—H20A	108.8	C35'—C36'—H36D	108.8
C19—C20—H20A	108.8	C37'—C36'—H36D	108.8
C21—C20—H20B	108.8	H36C—C36'—H36D	107.7
C19—C20—H20B	108.8	C38'—C37'—C36'	114.0 (7)
H20A—C20—H20B	107.7	C38'—C37'—H37C	108.8
N22—C21—C20	115.03 (15)	C36'—C37'—H37C	108.8
N22—C21—H21A	108.5	C38'—C37'—H37D	108.8
C20—C21—H21A	108.5	C36'—C37'—H37D	108.8
N22—C21—H21B	108.5	H37C—C37'—H37D	107.6
C20—C21—H21B	108.5	C37'—C38'—C39'	115.5 (8)
H21A—C21—H21B	107.5	C37'—C38'—H38C	108.4
C27—N22—C21	112.56 (14)	C39'—C38'—H38C	108.4
C27—N22—C23	108.83 (15)	C37'—C38'—H38D	108.4
C21—N22—C23	107.32 (14)	C39'—C38'—H38D	108.4
N22—C23—C24	110.88 (17)	H38C—C38'—H38D	107.5
N22—C23—H23A	109.5	C40—C39'—C38'	119.4 (8)
C24—C23—H23A	109.5	C40—C39'—H39C	107.5
N22—C23—H23B	109.5	C38'—C39'—H39C	107.5
C24—C23—H23B	109.5	C40—C39'—H39D	107.5
H23A—C23—H23B	108.1	C38'—C39'—H39D	107.5
O25—C24—C23	111.37 (19)	H39C—C39'—H39D	107.0
O25—C24—H24A	109.4	O42—C40—O41	124.03 (16)
C23—C24—H24A	109.4	O42—C40—C39'	129.0 (6)
O25—C24—H24B	109.4	O41—C40—C39'	104.8 (5)
C23—C24—H24B	109.4	O42—C40—C39	118.8 (2)
H24A—C24—H24B	108.0	O41—C40—C39	117.10 (19)
C24—O25—C26	108.91 (17)	C40—O41—H41	109.5
			
C9—N1—C2—N3	−0.2 (3)	N11—C12'—C13'—C14'	171.7 (16)
N1—C2—N3—C4	−0.1 (3)	C12'—C13'—C14'—C15'	1 (2)
C2—N3—C4—N11	−178.27 (15)	C12'—C13'—C14'—Cl1'	−168 (3)
C2—N3—C4—C10	1.1 (2)	C13'—C14'—C15'—C16'	−2 (5)
C10—C5—C6—O18	−179.65 (15)	Cl1'—C14'—C15'—C16'	171 (4)
C10—C5—C6—C7	−0.4 (2)	C13'—C14'—C15'—F1'	177 (6)
O18—C6—C7—O28	0.7 (2)	Cl1'—C14'—C15'—F1'	−10 (7)
C5—C6—C7—O28	−178.59 (15)	F1'—C15'—C16'—C17'	177 (3)
O18—C6—C7—C8	−179.53 (15)	C14'—C15'—C16'—C17'	−4 (6)
C5—C6—C7—C8	1.2 (3)	C15'—C16'—C17'—C12'	11 (6)
O28—C7—C8—C9	179.02 (15)	C13'—C12'—C17'—C16'	−11 (4)
C6—C7—C8—C9	−0.7 (2)	N11—C12'—C17'—C16'	−161 (4)
C2—N1—C9—C10	−0.5 (2)	C5—C6—O18—C19	−1.1 (2)
C2—N1—C9—C8	179.41 (16)	C7—C6—O18—C19	179.61 (14)
C7—C8—C9—N1	179.62 (15)	C6—O18—C19—C20	174.33 (14)
C7—C8—C9—C10	−0.5 (2)	O18—C19—C20—C21	166.04 (15)
N1—C9—C10—C5	−178.89 (15)	C19—C20—C21—N22	54.0 (2)
C8—C9—C10—C5	1.2 (2)	C20—C21—N22—C27	54.86 (19)
N1—C9—C10—C4	1.4 (2)	C20—C21—N22—C23	174.57 (15)
C8—C9—C10—C4	−178.49 (15)	C27—N22—C23—C24	−54.7 (2)
C6—C5—C10—C9	−0.7 (2)	C21—N22—C23—C24	−176.76 (17)
C6—C5—C10—C4	178.92 (16)	N22—C23—C24—O25	57.9 (2)
N3—C4—C10—C9	−1.7 (2)	C23—C24—O25—C26	−59.2 (2)
N11—C4—C10—C9	177.61 (15)	C24—O25—C26—C27	60.6 (2)
N3—C4—C10—C5	178.60 (16)	C21—N22—C27—C26	173.58 (15)
N11—C4—C10—C5	−2.1 (3)	C23—N22—C27—C26	54.74 (19)
N3—C4—N11—C12	−0.6 (3)	O25—C26—C27—N22	−59.3 (2)
C10—C4—N11—C12	−179.94 (18)	C8—C7—O28—C29	−3.9 (2)
N3—C4—N11—C12'	−3.5 (12)	C6—C7—O28—C29	175.88 (15)
C10—C4—N11—C12'	177.1 (11)	O31—C32—C33—C34	41.3 (3)
C4—N11—C12—C17	−29.1 (3)	O30—C32—C33—C34	−140.79 (17)
C4—N11—C12—C13	153.23 (19)	C32—C33—C34—C35'	−160.0 (6)
C17—C12—C13—C14	−1.3 (3)	C32—C33—C34—C35	−174.15 (19)
N11—C12—C13—C14	176.4 (2)	C33—C34—C35—C36	179.3 (2)
C12—C13—C14—C15	0.1 (3)	C34—C35—C36—C37	179.9 (2)
C12—C13—C14—Cl1	178.38 (17)	C35—C36—C37—C38	−178.5 (2)
C13—C14—C15—F1	−177 (2)	C36—C37—C38—C39	−177.9 (2)
Cl1—C14—C15—F1	5 (2)	C37—C38—C39—C40	−69.0 (3)
C13—C14—C15—C16	1.2 (4)	C33—C34—C35'—C36'	169.2 (6)
Cl1—C14—C15—C16	−177.0 (2)	C34—C35'—C36'—C37'	179.8 (8)
F1—C15—C16—C17	176.9 (19)	C35'—C36'—C37'—C38'	−67.5 (11)
C14—C15—C16—C17	−1.3 (4)	C36'—C37'—C38'—C39'	−169.1 (9)
C15—C16—C17—C12	0.1 (4)	C37'—C38'—C39'—C40	−179.1 (9)
C13—C12—C17—C16	1.2 (3)	C38'—C39'—C40—O42	−103.4 (10)
N11—C12—C17—C16	−176.4 (2)	C38'—C39'—C40—O41	60.0 (11)
C4—N11—C12'—C13'	158.5 (10)	C38—C39—C40—O42	−99.5 (3)
C4—N11—C12'—C17'	−49 (5)	C38—C39—C40—O41	83.0 (3)
C17'—C12'—C13'—C14'	5 (2)		

### Hydrogen-bond geometry (Å, º)

**Table:**

*D*—H···*A*	*D*—H	H···*A*	*D*···*A*	*D*—H···*A*
N11—H11···O42^i^	0.84 (2)	2.20 (2)	3.025 (2)	169.6 (18)
O41—H41···N22^ii^	1.01	1.78	2.6566 (19)	144
O30—H30···N1	0.99 (3)	1.65 (3)	2.6135 (19)	166 (2)
C5—H5···O42^i^	0.95	2.25	3.194 (2)	170
C2—H2···F1^iii^	0.95	2.15	3.07 (3)	163
C2—H2···F1′^iii^	0.95	2.32	3.253 (3)	166
C29—H29*B*···O18^iv^	0.98	2.65	3.6101 (19)	167
C23—H23*B*···O25^v^	0.99	2.57	3.220 (2)	123
C27—H27*B*···F1^vi^	0.99	2.71	3.68 (4)	166
C39—H39*A*···O30^vii^	0.99	2.50	3.255 (4)	133
C21—H21*B*···O31^viii^	0.99	2.29	3.234 (2)	160
C13—H13···O30^ix^	0.95	2.39	3.139 (3)	135
C13′—H13′···O30^ix^	0.95	2.53	3.268 (3)	135
*Cg*2···*Cg*2^viii^			3.5358 (11)	0 (1)
*Cg*2···*Cg*3^viii^			3.7909 (11)	1 (1)
*Cg*2···*Cg*3^ix^			3.7530 (11)	1 (1)
*Cg*3···*Cg*3^viii^			3.7934 (11)	0 (1)

Symmetry codes: (i) *x*−1/2, −*y*+3/2, *z*−1/2; (ii) *x*+1/2, −*y*+3/2, *z*+1/2; (iii) −*x*, −*y*+2, −*z*+1; (iv) −*x*+2, −*y*+1, −*z*+1; (v) −*x*+3/2, *y*−1/2, −*z*+1/2; (vi) *x*+1, *y*, *z*; (vii) −*x*+3/2, *y*+1/2, −*z*+3/2; (viii) −*x*+1, −*y*+1, −*z*+1; (ix) −*x*+1, −*y*+2, −*z*+1.
